# Development of a risk prediction model for left ventricular thrombosis in STEMI patients

**DOI:** 10.3389/fcvm.2026.1704792

**Published:** 2026-02-04

**Authors:** Jingjing Wang, Ping Ma, QingBin Xu, Lan Hai, Shichang Zhang

**Affiliations:** 1 Department of Critical Care Medicine, People's Hospital of Ningxia Hui Autonomous Region, Ningxia Medical University, Yinchuan, China; 2 Department of Cardiovascular Medicine, Cardiovascular and Cerebrovascular Disease Hospital of General Hospital of Ningxia Medical University, Yinchuan, China; 3 Department of Cardiovascular Medicine, The Fifth People's Hospital of Ningxia, Shizuishan, China

**Keywords:** acute ST-segment elevation myocardial infarction, left ventricular thrombus, nomogram, risk factors, risk prediction model

## Abstract

**Purpose:**

To develop a risk prediction model for left ventricular thrombus (LVT) formation in patients with acute ST-segment elevation myocardial infarction (STEMI).

**Patients and methods:**

We performed a retrospective analysis of patients with STEMI in our hospital between October 2017 to October 2020. According to transthoracic echocardiography, these patients were included in the LVT group (*n* = 50) or no-LVT group (*n* = 130). Clinical data were collected from both groups. The comparison between groups, single-factor logistic regression analysis, Lasso regression analysis and multi-factor logistic regression analysis were performed successively to screen the risk factors and to establish risk prediction models. After evaluation and internal verification, we obtained an optimal risk prediction model. A nomogram was constructed to visualize the optimal model.

**Results:**

The risk prediction model contained seven variables including MCV (OR = 1.251, 95% CI = 1.021–1.531, *P* = 0.030), D-Dimer (OR = 9.798, 95% CI = 2.630–36.503, *P* = 0.001), CRP (OR = 1.033, 95% CI = 1.011–1.055, *P* = 0.003), LVEF (OR = 0.903, 95% CI = 0.819–0.995, *P* = 0.040), A wave velocity (OR = 0.044, 95% CI = 0.002–0.906, *P* = 0.043), pericardial effusion (OR = 16.926, 95% CI = 2.767–103.522, *P* = 0.002) and anterior wall infarction (OR = 12.275, 95% CI = 2.136–70.548, *P* = 0.005). The C-index value was 0.966 and the area under the ROC curve was 96.6% (95% CI = 0.9399–0.9914). Simple cross-validation, k-fold, leave-one-out, and bootstrap analyses were used to internally verify the model. The accuracy of the model were 0.926, 0.9, 0.9, and 0.899, and Kappa values were 0.807, 0.744, 0.744, and 0.739. The area under the ROC curve was 94.7%, as verified by bootstrapping.

**Conclusion:**

MCV, D-dimer level, CRP level, LVEF, A-wave velocity, pericardial effusion, and anterior wall infarction were independently related to the occurrence of LVT in STEMI at the acute stage. The multivariate logistic regression risk prediction model developed in this study demonstrates good discrimination and calibration, enabling preliminary risk stratification for LVT in patients with acute STEMI.

## 1 Introduction

ST-segment elevation myocardial infarction (STEMI) is a clinical syndrome characterized by myocardial ischemia symptoms, persistent ST segment elevation and elevated myocardial injury markers (1). With the early revascularization such as thrombolysis and percutaneous transluminal coronary intervention (PCI), the mortality rate of patients decreases, but its complications, such as papillary muscle rupture, heart rupture and ventricular aneurysm, still affect the long-term prognosis of patients (2). LVT is one of the complications of STEMI (3). The formation of LVT is related to the classic Virchow's triad, that is, vascular endothelial injury, hypercoagulable state of blood and abnormal hemodynamics. These three preconditions of thrombosis are reflected to different degrees in patients with STEMI (4). It has been reported that, before thrombolytic therapy, the incidence of LVT was 54% (5), and the incidence of LVT decreased to 28% after thrombolysis (6). In recent years, with the improvement in early vascular recanalization technology and patient management, the incidence of LVT has further decreased, ranging from 0.7% to 8.4% (7, 8).

LVT can fall off the wall of the ventricle and reach various tissues and organs with blood flow, which obviously increases the risk of systemic embolism, ischemic stroke, and cardiovascular adverse events, and has a profound impact on the survival and prognosis of patients. It is reported that LVT is an independent predictor of major cardiovascular adverse events in one year, and patients with LVT have a higher incidence of major cardiovascular adverse events than those without LVT (21.7% vs. 10.3%) (9). Patients with LVT often have no characteristic clinical manifestations, which are mostly found in clinical routine echocardiography. They can also be found when infarctions in other organs occur, such as stroke, which may have caused serious adverse clinical outcomes. Therefore, it is extremely important to identify LVT-high-risk patients early and actively intervene clinically.

There have been many studies on the risk factors of LVT formation, and the reported risk factors include extensive anterior wall infarction, ventricular aneurysm, decreased left ventricular ejection fraction, and severe abnormal wall motion (7, 8, 10). However, at present, there is no recognized index with satisfactory prediction ability or a related risk prediction model. The risk prediction model based on logistic regression or COX regression provides a certain value for early identification, intervention, and prognosis evaluation of patients at high risk of some diseases in the clinic and is widely used in related research in medical and biological fields. In this study, based on LASSO regression and logistic regression, a risk prediction model of LVT in acute STEMI patients was established, and a nomogram was drawn to visualize the model for clinical use, which provided a reference for the clinical prediction of LVT.

## 2 Materials and methods

### 2.1 Patients and design

We retrospectively analyzed patients with STEMI at the Ningxia Medical University General Hospital and the Department of Cardiology Heart-Brain-Vascular Hospital between October 2017 and October 2020. The clinical data of the two groups were collected from the e*lectronic* medical record system. The diagnostic criteria for STEMI are at least two contiguous leads with ST-segment elevation ≥ 2.5 mm in men, ≥1.5 mm in women in leads V2–V3 and/or ≥1 mm in the other leads, and symptoms of myocardial ischemia or an increase and/or decrease in cTn values with at least one value above the 99th percentile URL. According to in-hospital transthoracic echocardiography, the patients were diagnosed with LVT. (Echocardiography showed an echo-dense mass in the left ventricle, which had a clear edge and was adjacent to the non-energetic myocardium. It could be clearly identified in the whole cardiac cycle, and could be distinguished from the muscle trabecula) (11). Patients with myocardial infarction, arterial or venous thrombosis, thrombotic hematological diseases, dilated cardiomyopathy and severe valve diseases in the past were excluded.

### 2.2 Variable extraction

The clinical data of the two groups were collected from the *electronic* medical record system. Information on the following indices was extracted: baseline data (including age, sex, body mass index, blood pressure at admission, and basic diseases such as hypertension, diabetes, abnormal lipid metabolism, smoking history, and drinking history), laboratory data (blood routine, biochemical indexes, coagulation indices, thyroid function, CRP, glycosylated hemoglobin, NT-Pro BNP), echocardiographic data (LVEDD, LVEDS, LVEF, LVFS, abnormal regional wall motion, pericardial effusion, left ventricular aneurysm, mitral regurgitation, and echocardiographic diagnosis of LVT from the onset time of chest pain), PCI, and other information (location of criminal blood vessels, number of criminal blood vessels, infarct location, and Killip grade at admission).

### 2.3 Statistical analysis

The collected data were sorted and statistically analyzed using SPSS 25.0, and R version 4.0.4. Continuous variables are expressed as mean ± standard deviation (x bar ± SD), and normality tests were performed. The t-test was used for distributed continuous variables, and the nonparametric rank-sum test was used for non-normally distributed continuous variables to compare the differences between groups. Categorical data are expressed as frequencies (percentages), and the chi-square test was used to compare differences between groups. *P* < 0.05 was considered statistically significant. The univariate logistic regression, Lasso logistic regression and multivariate logistic regression were successively performed to preliminarily establish two logistic regression models. Internal evaluation and verification were performed using the two established risk prediction models to obtain the optimal risk prediction model. Based on the optimal model, a nomogram was drawn to visualize the optimal model.

## 3 Results

There were about 3,096 cases with STEMI and 50 were diagnosed with LVT. The 50 patients diagnosed with LVT were included in the case group. And we Use the SORTBY and RANDARRAY functions in Excel to randomly select 130 STEMI patients without LVT as the control group.

### 3.1 Comparison of clinical date between groups

The case group included 50 patients and the control group included 130 patients. There were no significant differences in age, sex, body mass index, blood pressure at admission, hypertension, diabetes, dyslipidemia, hyperhomocysteinemia, smoking, or drinking history between the two groups. Mean corpuscular volume (MCV), mean corpuscular hemoglobin concentration (MCHC), red cell distribution width (RDW), prothrombin time (PT), prothrombin time activity (PTA), D-Dimer, international normalized ratio (INR), fibrinogen (FIB), C-reactive protein (CRP), N-terminal Pro-B type natriuretic peptide (NT-Pro BNP), left ventricular end-diastolic dimension (LVEDD), left ventricular end-systolic dimension (LVESD), left ventricular ejection fraction (LVEF), LVEF <40%, left ventricular fraction shortening (LVFS), A-wave velocity, E-wave velocity, E/A <1, abnormal wall motion, pericardial effusion, left ventricular aneurysm, percutaneous transluminal coronary intervention (PCI), left anterior descending branch lesion, anterior wall infarction and Killip grade II-IV at admission showed significant differences between the two groups (*P* < 0.05) (Table 1).

**Table 1:** Comparison of clinical data between groups.

Characteristics and variables	Lvt (*n* = 50)	no-lvt (*n* = 130)	*P*
Age (years)	62.24 ± 13.58	60.66 ± 12.58	0.530
BMI (kg/m^3^)	24.47 ± 3.11	24.81 ± 4.48	0.640
SBP (mmHg)	118 ± 21	124 ± 19	0.065
DBP (mmHg)	75 ± 15	76 ± 15	0.620
Male, *n* (%)	37 (74%)	107 (82%)	0.212
Hypertension, *n* (%)	25 (50%)	60 (46%)	0.643
Diabetes, *n* (%)	10 (18%)	27 (20%)	0.909
Dyslipidemia, *n* (%)	8 (16%)	38 (29.2%)	0.068
Hyperhomocysteinemia, *n* (%)	14 (28%)	21 (16.2%)	0.072
Smoke, *n* (%)	31 (62%)	82 (63.1%)	0.894
Drink, *n* (%)	5 (10%)	27 (20.8%)	0.091
WBC (10^9^/L)	10.72 ± 5.41	10.21 ± 2.91	0.437
NEUT# (10^9^/L)	8.54 ± 5.07	8.06 ± 3.02	0.622
LYM# (10^9^/L)	1.43 ± 0.62	1.46 ± 0.69	0.964
MCV (fL)	92.05 ± 5.70	89.91 ± 4.90	0.003
MCHC (g/L)	341.18 ± 13.53	347.46 ± 12.54	0.007
RDW (fL)	43.92 ± 3.47	41.16 ± 4.48	<0.001
Platelet (10^9^/L)	234.32 ± 89.98	218.38 ± 56.69	0.816
Procalcitonin (ng/mL)	0.26 ± 0.16	0.23 ± 0.05	0.903
MPV (fL)	10.36 ± 0.91	10.47 ± 0.90	0.598
PDW (fL)	12.51 ± 5.11	12.37 ± 3.43	0.563
PLCR (%)	27.61 ± 7.35	28.65 ± 7.48	0.456
Albumin (g/L)	37.60 ± 4.82	39.61 ± 4.06	0.057
Globulin (g/L)	28.76 ± 4.81	28.63 ± 3.94	0.912
A/G	1.34 ± 0.27	9.61 ± 4.06	0.091
Creatinine (*μ*mol/L)	87.78 ± 64.57	71.81 ± 20.09	0.100
Urea nitrogen (mmol/L)	6.649 ± 5.13	5.76 ± 1.87	0.786
Uric acid (μmol/L)	360 ± 103.89	346.20 ± 85.78	0.559
AST (U/L)	224.64 ± 261.78	139.61 ± 131.21	0.585
ALT (U/L)	84.10 ± 124.49	51.02 ± 24.47	0.862
PT (s)	12.97 ± 4.03	12.19 ± 4.07	0.015
PTA (%)	87.66 ± 20.72	97.67 ± 16.58	0.001
INR	1.11 ± 0.33	1.04 ± 0.35	0.003
D-Dimer (mg/L)	0.46 ± 0.40	1.95 ± 2.87	<0.001
FIB (g/L)	3.70 ± 1.65	3.16 ± 1.18	0.039
CRP (mg/L)	49.86 ± 55.14	15.59 ± 23.07	<0.001
HCY (μmol/L)	31.64 ± 27.83	24.70 ± 16.10	0.116
NT-Pro BNP (pg/mL)	4,892.72 ± 8,548.02	1,140.10 ± 1,977.02	<0.001
TSH (μIU/mL)	1.45 ± 1.21	1.59 ± 1.51	0.506
LVEDD (mm)	54.94 ± 4.96	51.05 ± 4.68	<0.001
LVESD (mm)	42.52 ± 5.09	36.61 ± 5.82	<0.001
LVSV (mL)	65.57 ± 14.55	67.50 ± 16.90	0.821
LVEF (%)	44.95 ± 7.44	54.25 ± 9.87	<0.001
LVFS (%)	22.75 ± 4.63	28.89 ± 7.54	<0.001
E wave (m/s)	0.58 ± 0.18	0.64 ± 0.17	0.026
A wave (m/s)	0.70 ± 0.21	0.80 ± 0.16	<0.001
E/A	0.97 ± 0.66	0.85 ± 0.37	0.706
E/A < 1, *n* (%)	39 (78%)	57 (44%)	0.007
PE, *n* (%)	20 (38%)	6 (4.8%)	<0.001
LVA, *n* (%)	14 (20%)	8 (4.8%)	0.002
LVEF < 40%, *n* (%)	13 (70%)	5 (3.8%)	<0.001
AVWM, *n* (%)	48 (96%)	98 (75%)	0.001
MR, *n* (%)	32 (60%)	78 (64%)	0.622
PCI, *n* (%)	44 (88%)	125 (96.2%)	0.041
Number of lesions			0.072
SVD, *n* (%)	27 (64%)	57 (46%)	
DVD, *n* (%)	6 (14%)	30 (24%)	
TVD, *n* (%)	9 (22%)	38 (30%)	
LAD, *n* (%)	41 (93%)	99 (79%)	0.037
Ant-STEMI, *n* (%)	47 (94%)	59 (45%)	<0.001
Killip II-IV grade, *n* (%)	24 (48%)	21 (16%)	<0.001

BMI, body mass index; SBP, systolic blood pressure; DBP, diastolic blood pressure; WBC, white blood cell count; NEUT#, neutrophil count; LYM#, lymphocyte count; MCV, mean corpuscular volume; MCHC, mean corpuscular hemoglobin concentration; RDW, Red cell distribution width; MPV, mean platelet volume; PDW, platelet distribution width; PLCR, platelet larger cell ratio; A/G, ratio of albumin to globulin; AST, aspartate aminotransferase; ALT, alanine aminotransferase; PT, prothrombin time; PTA, prothrombin time activity; INR, international normalized ratio; FIB, fibrinogen; CRP, C-reactive protein; HCY, homocysteine; NT-Pro BNP, N-terminal Pro-B type natriuretic peptide; TSH, thyroid-stimulating hormone; LVEDD, left ventricular end-diastolic dimension; LVESD, left ventricular end-systolic dimension; LVSV, left ventricular stroke volume; LVEF, left ventricular ejection fraction; LVFS, left ventricular fraction shortening; E/A, ratio of E-wave to A-wave; PE, Pericardial effusion; LVA, left ventricular aneurysm; AVWM, abnormal ventricular wall motion; MR, mitral regurgitation; PCI, percutaneous transluminal coronary intervention; SVD, Single vessel disease; DVD, double vessel disease; TVD, triple vessel disease; LAD, Left anterior descending branch disease; Ant-STEMI, anterior wall ST-segment elevation myocardial infarction.

### 3.2 Univariate logistic regression

The results of the univariate logistic regression analysis showed that 19 variables, including MCV, MCHC, RDW, D-dimer, FIB, CRP, NT-pro BNP, LVEDD, LVESD, LVEF, LVEF < 40%, LVFS, A wave velocity, pericardial effusion, left ventricular aneurysm, abnormal wall motion, anterior wall infarction, left anterior descending artery disease, and Killip classification II–IV on admission, were associated with LVT formation in STEMI patients at the acute stage (Table 2).

**Table 2:** The result of univariate logistic regression.

Variables	*β*	Wald	OR	95% CI	*P*
Male	0.491	1.540	1.635	(0.752–3.552)	0.215
Age	0.008	0.399	1.008	(0.983–1.034)	0.528
BMI	0.036	0.896	1.037	(0.896–1.039)	0.344
SBP	0.016	3.447	1.017	(0.999–1.034)	0.063
DBP	0.006	0.264	1.006	(0.984–1.028)	0.607
Hypertension	0.154	0.214	1.167	(0.607–2.241)	0.644
Diabetes	0.047	0.013	0.954	(0.423–2.149)	0.909
Dyslipidemia	0.774	3.221	2.168	(0.931–5.050)	0.073
Hyperhomocysteinemia	0.702	3.162	2.019	(0.931–4.378)	0.075
Smoke	0.046	0.018	1.047	(0.534–2.052)	0.894
Drink	0.904	3.055	2.471	(0.896–6.812)	0.080
WBC	0.035	0.644	1.035	(0.951–1.126)	0.422
NEUT#	0.034	0.593	1.035	(0.949–1.128)	0.441
LYM#	0.090	0.127	1.094	(0.668–1.791)	0.722
MCV	0.093	6.141	1.098	(1.020–1.182)	0.013
MCHC	−0.039	7.699	0.961	(0.935–0.989)	0.006
RDW	0.242	16.649	1.274	(1.134–1.431)	<0.001
platelet	0.003	1.952	1.003	(0.999–1.008)	0.162
MPV	0.134	0.497	1.143	(0.788–1.658)	0.481
Procalcitonin	−3.224	2.579	0.040	(0.001–2.036)	0.108
PDW	0.009	0.045	1.009	(0.932–1.092)	0.831
PLCR	−0.019	0.697	0.981	(0.938–1.026)	0.404
Albumin	0.109	7.342	1.115	(1.031–1.207)	0.057
Globulin	−0.008	0.037	0.847	(0.918–1.073)	0.847
A/G	1.323	3.099	3.756	(0.861–16.391)	0.078
PT	0.042	1.117	0.959	(0.888–1.036)	0.290
PTA	0.030	9.470	1.031	(1.011–1.051)	0.052
INR	−0.576	1.372	0.562	(0.214–1.474)	0.562
D-Dimer	1.922	28.236	6.832	(3.363–13.880)	<0.001
FIB	0.287	5.570	1.333	(1.050–1.692)	0.018
TSH	0.074	0.126	1.077	(0.841–1.379)	0.559
CRP	0.022	15.883	1.022	(1.011–1.034)	<0.001
NT-Pro BNP	<0.001	11.573	1.000	(1.000–1.000)	0.001
LVEDD	0.168	18.881	1.184	(1.097–1.277)	<0.001
LVESD	0.185	25.867	1.203	(1.120–1.291)	<0.001
LVSV	0.008	0.509	1.008	(0.987–1.029)	0.475
LVEF	−0.138	26.377	0.871	(0.827–0.918)	<0.001
LVFS	−0.193	22.078	0.824	(0.760–0.893)	<0.001
E WAVE	2.031	3.913	7.619	(1.019–56.982)	0.058
A WAVE	−3.176	9.820	0.042	(0.006–0.304)	0.002
E/A	−0.483	2.074	0.617	(0.32–1.19)	0.150
E/A<1	1.513	15.491	4.541	(2.137–9.646)	0.052
LVEF < 40%	2.173	15.136	8.784	(2.939–26.248)	<0.001
PE	2.623	26.662	13.778	(5.091–37.290)	<0.001
LVA	1.780	13.635	5.931	(2.305–15.256)	<0.001
MR	0.150	0.190	1.162	(0.591–2.287)	0.663
Ant-STEMI	3.222	26.81	25.067	(7.404–84.859)	<0.001
AVWM	2.473	11.011	11.862	(2.752–51.124)	0.001
PCI	−1.226	3.785	0.293	(0.085–1.009)	0.052
LAD	1.316	4.276	3.727	(1.071–12.972)	0.039
Killip II-IV	1.988	27.269	7.304	(3.463–15.407)	<0.001

BMI, body mass index; SBP, systolic blood pressure; DBP, diastolic blood pressure; WBC, white blood cell count; NEUT#, neutrophil count; LYM#, lymphocyte count; MCV, mean corpuscular volume; MCHC, mean corpuscular hemoglobin concentration; RDW, red cell distribution width; MPV, mean platelet volume; PDW, platelet distribution width; PLCR, platelet larger cell ratio; A/G, ratio of albumin to globulin; PT, prothrombin time; PTA, prothrombin time activity; INR, international normalized ratio; FIB, fibrinogen; TSH, thyroid-stimulating hormone; CRP, C-reactive protein; NT-Pro BNP, N-terminal Pro-B type natriuretic peptide; LVEDD, left ventricular end-diastolic dimension; LVESD, left ventricular end-systolic dimension; LVSV, left ventricular stroke volume; LVEF, left ventricular ejection fraction; LVFS, left ventricular fraction shortening; E/A, ratio of E-wave to A-wave; PE, pericardial effusion; LVA, left ventricular aneurysm; MR, mitral regurgitation; Ant-STEMI, anterior wall ST-segment elevation myocardial infarction; AVWM, abnormal ventricular wall motion; PCI, percutaneous transluminal coronary intervention; LAD, Left anterior descending branch disease.

### 3.3 Construction of the risk prediction model

#### 3.3.1 Lasso logistic regression

**Figure 1:**
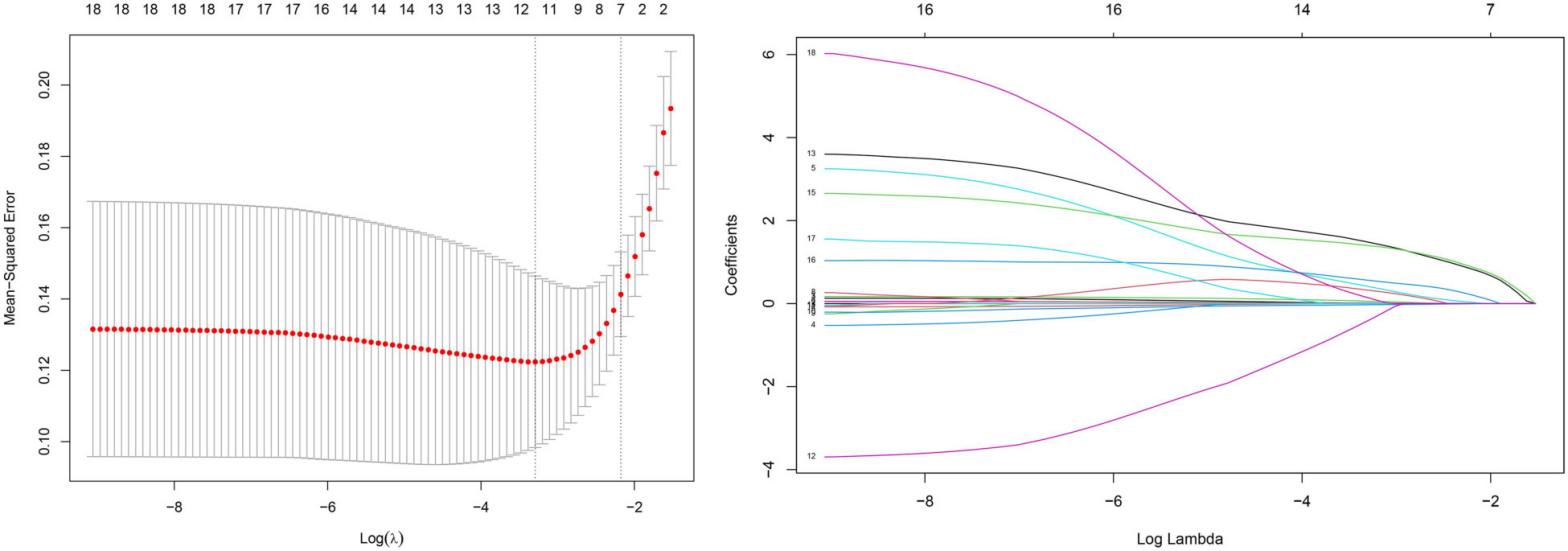
Variable screening based on lasso regression. (left) Characterization of the variation of variable coefficients; (right) The process of selecting the optimal value of the parameter *λ* in the Lasso regression model by the cross-validation method.

The LASSO regression for the 19 variables and the cross-validation of the LASSO regression results for all lambda values was shown in Figure 1. The left dashed line is the *λ* value with the smallest MSE, and the right dashed line is the *λ* value corresponding to the combination of variables with the least MSE in the variance range. According to the two *λ* values, the regression coefficient of each variable after regularization can be obtained, in which some variable coefficients are compressed to zero, and the variables with different coefficients corresponding to the two *λ* values are retained. Two variable combinations were formed, which are represented by combinations 1 and 2.

Combination 1 contained 12 variables (MCV, MCHC, RDW, D-dimer, CRP, LVEF, A wave, PE, LVA, ANT-STEMI, Killip II-IV, and LVEF < 40%), and combination 2 contained six variables (D-dimer, CRP, LVEF, PE, ANT-STEMI, and Killip II–IV).

#### 3.3.2 Multivariate logistic regression model construction

Multivariate logistic regression analysis was performed on two variable combinations. The results of multivariate logistic regression analysis in combination 1 showed that MCV (OR = 1.251, 95% CI = 1.021–1.531, *P* = 0.030), D-Dimer (OR = 9.798, 95% CI = 2.630–36.503, *P* = 0.001), CRP (OR = 1.033, 95% CI = 1.011–1.055, *P* = 0.003), LVEF (OR = 0.903, 95% CI = 0.819–0.995, *P* = 0.040), A wave velocity (OR = 0.044, 95% CI = 0.002–0.906, *P* = 0.043), pericardial effusion (OR = 16.926, 95% CI = 2.767–103.522, *P* = 0.002), and anterior wall infarction (OR = 12.275, 95% CI = 2.136–70.548, *P* = 0.005) were independently related to the occurrence of LVT in STEMI patients (Table 3).

**Table 3:** The results of multivariate logistic regression analysis in combination 1.

Variables	β	Walds	OR	95% CI	*P*
MCV	0.224	4.682	1.251	1.021–1.531	0.030
MCHC	−0.040	1.397	0.961	0.900–1.026	0.237
RDW	0.089	0.322	1.093	0.804–1.486	0.570
D-dimer	2.282	11.566	9.798	2.630–36.503	0.001
CRP	0.032	8.721	1.033	1.011–1.055	0.003
LVEF	−0.102	4.219	0.903	0.819–0.995	0.040
A wave	−3.127	4.097	0.044	0.002–0.906	0.043
PE	2.829	9.374	16.926	2.767–103.522	0.002
LVA	0.708	0.618	2.030	0.347–11.871	0.432
ANT-STEMI	2.508	7.899	12.275	2.136–70.548	0.005
Killip II-IV	1.369	3.261	3.931	0.890–17.363	0.071
LVEF < 40%	0.924	0.403	2.519	0.146–43.596	0.525

MCV, mean corpuscular volume; MCHC, mean corpuscular hemoglobin concentration; RDW, red cell Distribution Width; CRP, C-reactive protein; LVEF, left ventricular ejection fraction; PE, pericardial effusion; LVA, left ventricular aneurysm; MR, mitral regurgitation; Ant-STEMI, anterior wall ST-segment elevation myocardial infarction.

The results of multivariate logistic regression analysis in combination 2 showed that D-Dimer (OR = 7.154, 95% CI = 2.504–20.438, *P* < 0.001), CRP (OR = 1.022, 95% CI = 1.007–1.037, *P* = 0.005), LVEF (OR = 0.917, 95% CI = 0.854–0.986, *P* = 0.018), pericardial effusion (OR = 9.476, 95% CI = 2.266–39.634, *P* = 0.002) and anterior wall infarction (OR = 12.32, 95% CI = 2.71–55.998, *P* = 0.001) were independently related to the occurrence of LVT in STEMI patients (Table 4).

**Table 4:** The results of multivariate logistic regression analysis in combination 2.

Variables	β	Walds	OR	95% CI	*P*
D-Dimer	1.968	13.498	7.154	2.504–20.438	<0.001
CRP	0.022	8.016	1.022	1.007–1.037	0.005
LVEF	−0.086	5.564	0.917	0.854–0.986	0.018
PE	2.249	9.488	9.476	2.266–39.634	0.002
ANT-STEMI	2.511	10.567	12.32	2.71–55.998	0.001
Killip II-IV	0.894	2.096	2.445	0.729–8.201	0.148

CRP, C-reactive protein; LVEF, left ventricular ejection fraction; PE, pericardial effusion; Ant-STEMI, anterior wall ST-segment elevation myocardial infarction.

According to the logistic regression analysis of the combination of the two variables, two logistic regression risk prediction models were established: Models 1 and 2. Model 1 contained seven variables: MCV, D-dimer, CRP, EF, A-wave velocity, pericardial effusion, and anterior wall infarction, while model 2 contained five variables: D-dimer, CRP, LVEF, pericardial effusion, and anterior wall infarction.

### 3.4 Optimal model selection

#### 3.4.1 Internal evaluation

**Figure 2:**
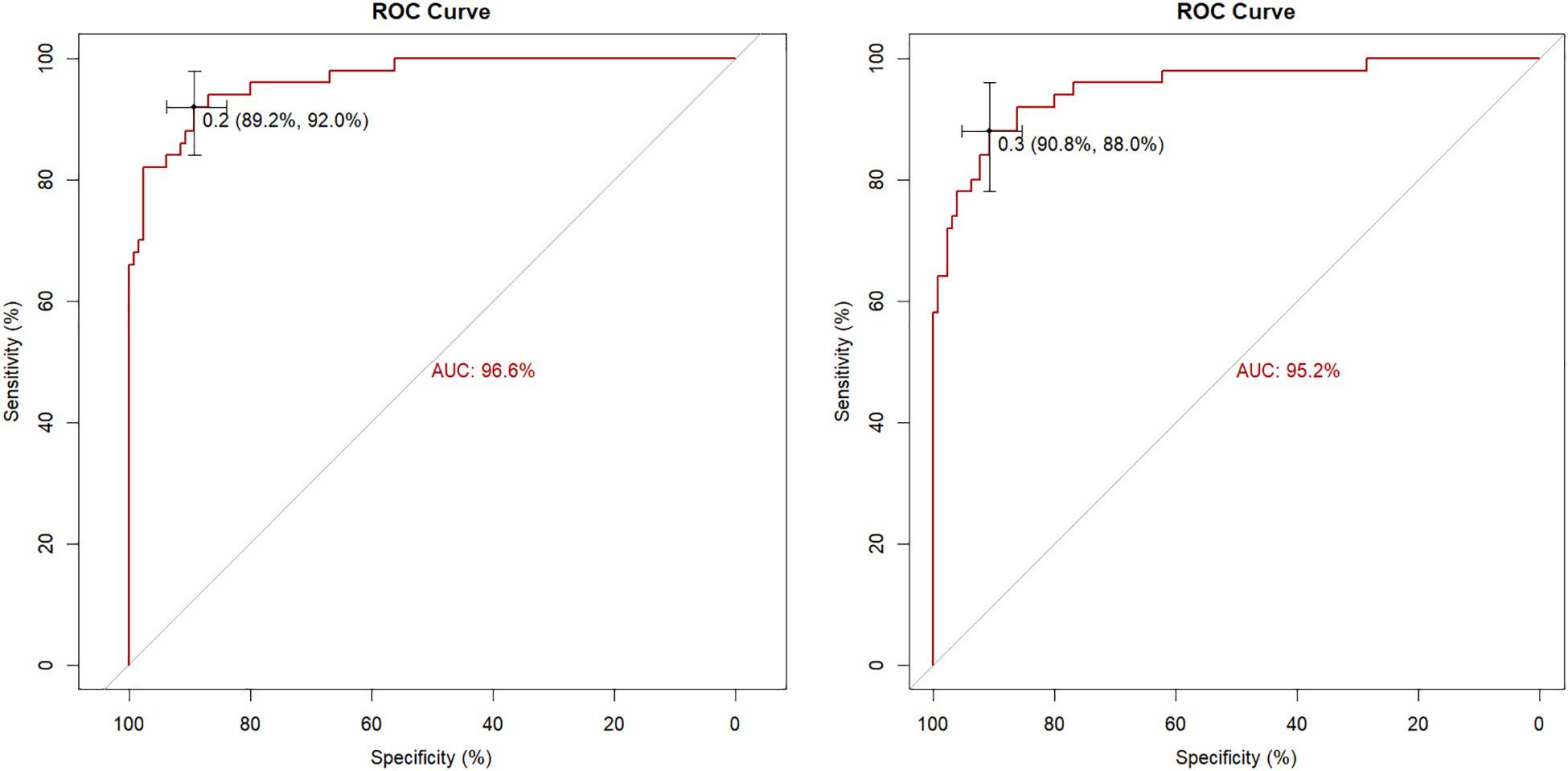
The ROC curve of the nomogram in the model 1(left)and model 2(right).

Internal evaluation and verification of the two risk prediction models were performed. The internal evaluations included discrimination and calibration. The C-index value of Model 1 was 0.966, and the area under the ROC curve was 96.6% (95% CI = 0.9399–0.9914. The C-index value of Model 2 was 0.952, and the area under the ROC curve was 95.2% (95% CI = 0.9158–0.9873 (Figure 2).

**Figure 3:**
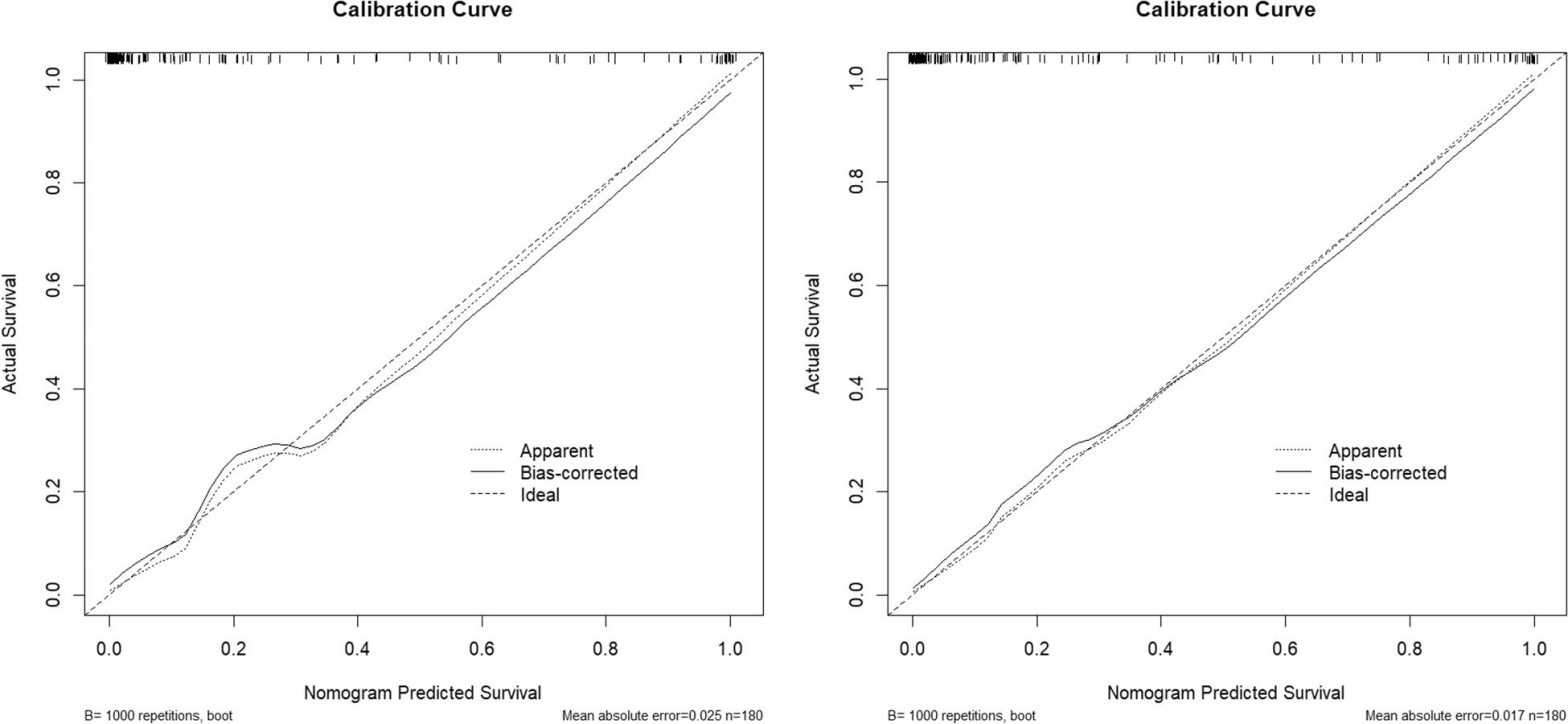
Calibration curve for the model 1(left) and the model 2 (right).

The calibration curves of the two models were drawn using the R software. The predicted results of the two models are in good agreement with those of the ideal model (Figure 3).

#### 3.4.2 Internal verification

Simple cross-validation, k-fold, leave-one-out, and bootstrap analyses were used to internally verify the model. The accuracy of two models were 0.926, 0.9, 0.9, 0.899 and 0.889, 0.889, 0.894, 0.886, and Kappa values were 0.807, 0.744, 0.744, 0.739 and 0.698, 0.716, 0.723, 0.704, respectively. The areas under the ROC curves of the two models were 94.7% and 92.8%, respectively, as verified by the bootstrap analysis (Table 5).

**Table 5:** Result of internal verification.

Method	Model 1		Model 2	
	Accuracy	kappa	Accuracy	kappa
Simple cross-validation	0.926	0.807	0.889	0.698
k-fold	0.9	0.744	0.889	0.716
leave-one-out	0.9	0.744	0.894	0.723
bootstrap	0.899	0.739	0.886	0.704

Through internal evaluation and verification of the two models, we believe that the prediction ability of Model 1 is better than that of Model 2, and Model 1 is taken as the optimal risk prediction model. The variables included in the optimal model were MCV, D-dimer, CRP, LVEF, A-wave velocity, anterior wall infarction, and pericardial effusion.

### 3.5 Nomogram for optimal model

Before drawing the nomogram, we calculated the optimal cut-off value for continuous variables. ROC curves of five continuous variables in model 1, such as red blood cell distribution width, D-dimer, CRP, LVEF, and A-wave velocity, were drawn using SPSS. The Jordan index of the best cut-off value of each variable was calculated, and the best cut-off value and the corresponding specificity and sensitivity of the five continuous variables were obtained (Table 6).

**Table 6:** Optimal cut-off value of continuous variables.

Variables	Cut-off value	Sensitivity	Specificity	Youden Index
MCV (fL)	89.85	0.74	0.546	0.286
D-Dimer (mg/L)	0.66	0.76	0.81	0.57
A wave (m/s)	0.74	0.64	0.354	0.286
CRP (mg/L)	11.6	0.8	0.54	0.34
LVEF (%)	51.28	0.92	0.477	0.443

MCV, mean corpuscular volume; CRP, C-reactive protein; LVEF, left ventricular ejection fraction.

**Figure 4:**
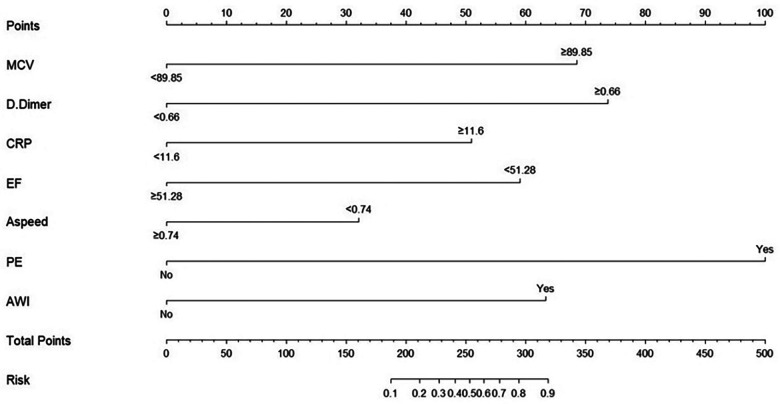
Nomogram of risk prediction model for LVT in patients with STEMI.

According to the best cutoff value, the continuous variables were converted into binary variables, and the nomogram of the best risk prediction model was drawn using the R software, which visualizes the risk prediction model and is convenient for clinical application (Figure 4).

## 4 Discussion

Prior to the widespread adoption of percutaneous coronary intervention (PCI), the incidence of left ventricular thrombus (LVT) in patients with ST-segment elevation myocardial infarction (STEMI) was considerably high. For instance, an Italian study published in 1986 reported an LVT incidence of 54% (5)—diagnosed by two-dimensional echocardiography—in STEMI patients who received neither anticoagulant nor antiplatelet therapy. Similarly, a 1989 Italian study found that among patients with anterior STEMI treated with thrombolysis, the LVT incidence was 28% (6). With the advent of early reperfusion strategies—particularly primary PCI—and the routine use of potent antithrombotic and anticoagulant therapies, the incidence of LVT has declined substantially. In a recent multicenter study using cardiac magnetic resonance (CMR) imaging, LVT was detected in 26 of 738 STEMI patients, yielding an overall incidence of 3.5%, with a higher rate of 7.1% observed specifically in those with anterior STEMI (12). Notably, CMR is currently regarded as the gold standard for LVT diagnosis due to its superior sensitivity and specificity. In contrast, transthoracic echocardiography (TTE)—the modality used in our study—may underestimate the true prevalence of LVT, potentially introducing bias in the sensitivity of prediction models based on TTE findings. Nevertheless, TTE remains the most widely used clinical tool for LVT screening owing to its accessibility, low cost, and feasibility at the bedside—advantages that are especially valuable in resource-limited settings. Our institution is located in an economically underdeveloped region where CMR is rarely utilized in routine practice due to its high cost, prolonged acquisition time, and the logistical challenges of transporting critically ill patients. Consequently, we elected to use TTE as the diagnostic criterion for LVT in this study to enhance the real-world relevance and preliminary translational potential of our findings. Among the 3,096 eligible STEMI patients included in our analysis, TTE identified LVT in 50 cases, corresponding to an incidence of 1.61%. This figure falls within the range of 0.7% (7) to 8.4% (8) reported in studies from the past decade that also relied on TTE for LVT diagnosis, supporting the consistency of our results with contemporary literature. In summary, advances in reperfusion therapy and antithrombotic management have markedly reduced the incidence of LVT in STEMI patients.

Despite the declining incidence, left ventricular thrombus (LVT) persists as a clinically significant complication of ST-elevation myocardial infarction (STEMI), adversely affecting long-term outcomes. However, there is a lack of effective predictive indicators to help clinicians identify high-risk patients early. In this study, we found that MCV, D-dimer level, CRP level, LVEF, A-wave velocity, pericardial effusion, and anterior wall infarction were independent risk factors for LVT.

The MCV is an index used to measure the volume of red blood cells and is mainly used in the differential diagnosis of anemia. In recent years, some studies have suggested that MCV is related to the mortality of patients with end-stage renal disease (13), all-cause mortality of acute heart failure (14), severity of coronary artery disease (15), adverse outcomes after PCI (16), and endothelial cell function (17), but the specific mechanism remains unclear. The research results of Yalcin Solak et al. show that endothelial function is negatively linearly correlated with MCV, revealing that endothelial dysfunction may be the internal mechanism between MCV and the occurrence of complex cardiovascular events in patients with end-stage renal disease (17). The research results of Wang Huaiyu showed that MCV is positively correlated with Gensini score, suggesting that MCV is significantly correlated with the severity of coronary artery disease (15); Masahiro Myojo et al. thought that risk stratification based on MCV may be valuable for evaluating the prognosis of patients after PCI (16); Tomaueda et al. think that MCV > 100 fl is an independent predictor of all-cause cardiovascular death in ADHF patients (18). The possible mechanisms of MCV and the above cardiovascular events include inflammatory reaction, malnutrition, abnormal bone marrow function, endothelial dysfunction, oxidative stress (13, 17), but further exploration is needed. However, there have been no reports of MCV related to thrombosis. The results of the comparison between the groups in this study suggested that the MCV in the case group was significantly higher than that in the control group. Multivariate regression analysis suggested that there is an independent correlation between them, which may suggest that patients with LVT have more severe vascular disease, more serious inflammatory and oxidative stress reactions, and more significant endothelial dysfunction. However, this hypothesis requires further investigation.

D-dimer is a soluble degradation product of fibrin, which is a product of cross-linked fibrin degraded by fibrinolytic enzyme (19). It is often used as a marker of activation of coagulation and fibrinolysis systems in clinics and is widely used in the diagnosis of venous thromboembolism (VTE). In recent years, it has been found that the D-dimer level in patients with acute coronary syndrome is closely related to the cardiac function and prognosis of patients after PCI (20). In a previous study, it was found that, in patients with left ventricular dysfunction after PCI, the elevation of D-dimer level was an independent predictor of left ventricular wall thrombus (21). Similarly, our study found that D-dimer was associated with LVT formation in STEMI patients, regardless of whether patients had cardiac dysfunction or whether they underwent PCI.

CRP is a sensitive non-specific inflammatory response marker in acute inflammation, which is mainly synthesized in the liver (22). Its transcription level is regulated by the cytokine interleukin-6, and its level can be increased 1,000 times when acute inflammation occurs (22). CRP is also considered an independent biomarker of cardiovascular risk (23). A large number of studies have shown that plasma CRP is not only involved in anti-inflammatory and pro-inflammatory processes, but is also related to endothelial dysfunction, leukocyte activation, angiogenesis, and other processes, which promote the pathogenesis of atherosclerosis, and can also induce platelet aggregation and activation and participate in thrombosis (24). It has been found that the change rate of CRP 24 h after admission is independently related to microvascular embolism and early left ventricular dysfunction after STEMI (25). The results of this study show that CRP level is independently related to the formation of LVT in STEMI patients, which is consistent with previously reported results (25).

Many studies have reported a correlation between LVEF and LVT in STEMI patients, and the LVEF decrease was independently related to LVT occurrence, which has been confirmed in many studies (7, 8, 10, 26). Our study results were consistent with those of previous studies, but the best cutoff value of LVEF for predicting LVT occurrence was different. Some studies suggest that LVEF <40% is an independent risk factor for LVT occurrence in acute extensive anterior wall or extensive anterior wall STEMI patients (26). Niazi et al. think that LVT is more common in patients with acute myocardial infarction whose ejection fraction is less than 30% (8). The different LVEF cutoff values may be related to sample selection, because different infarct sites have different effects on patients' cardiac function. Determining the best cutoff value of LVEF for predicting LVT formation requires a large-sample, multicenter clinical study.

E-wave and A-wave represent the mitral valve blood flow velocity in early and late diastole, respectively, and are often used to evaluate left ventricular diastolic function (27). In this study, the comparison between groups showed that the A-wave velocity and E-wave velocity in the case group were significantly lower than those in the control group (0.70, 0.21 vs. 0.80, 0.16, *P* < 0.001, 0.58, 0.18 vs. 0.64, 0.17, *P* = 0.026), and there was no statistical difference between the E/A groups. Multiple logistic regression analysis showed that the A-wave velocity was independently related to LVT formation in STEMI patients and that the diastolic function of patients with left ventricular thrombosis decreased more obviously than that of the control group. This result is also supported by previous studies. Pankaj Garg et al. conducted a prospective study by using four-dimensional blood flow cardiovascular magnetic resonance imaging and studied the related factors of LVT formation in STEMI patients by calculating the magnitude of the decrease of ventricular kinetic energy (KE) in different periods. The results showed that the decrease in A-wave KE from the middle of the ventricle to the apex of the heart in patients with LVT was significantly higher than that in patients without LVT (87% vs. 78%, *P* < 0.01), which suggests that late diastolic blood flow filling velocity plays an important role in left ventricular apical scour (28).

In previous studies, LVT was found to be more likely to occur in patients with anterior wall infarction and extensive anterior wall infarction. Therefore, in our study, the infarct site was divided into the anterior and non-anterior walls for comparison between the groups, and the difference was statistically significant. Logistic regression analysis showed that anterior wall infarction was independently related to LVT in patients with STEMI, consistent with previous research results (7, 8, 29).

This study found that there were significantly more patients with pericardial effusion in the case group than in the control group, and this difference was statistically significant. The number of patients with pericardial effusion ranged from low to moderate. No large pericardial effusions were observed. Pericardial effusion is a common complication in STEMI patients. A moderate–large amount of pericardial effusion often indicates more serious myocardial infarction, and the incidence of MACE increases significantly within one year after STEMI, which is a sign of poor prognosis (30). Jiang Yongxin et al. found that pericardial effusion was related to LVT after AMI by univariate analysis, but further multivariate analysis failed to prove that pericardial effusion was independently related to LVT formation (7). However, our study suggests that pericardial effusion is independently related to the occurrence of LVT, and eventually becomes an important index in the risk prediction model.

Based on the results of multivariate regression analysis, we established a multivariate regression model to predict the occurrence of left ventricular mural thrombus. The model contained these seven indicators, which were independently related to the occurrence of LVT. These seven indicators are easy to obtain in clinical practice with strong repeatability and low acquisition costs. For convenience of clinical use, we drew the nomogram of model 1 in R software, which can simply and quickly calculate the total score of each patient and evaluate the risk of LVT formation. Through internal evaluation and internal verification, the prediction efficiency of the model was found to be satisfactory.

## 5 Conclusion

MCV, D-dimer level, CRP level, LVEF, A-wave velocity, pericardial effusion, and anterior wall infarction were independently related to the occurrence of LVT in STEMI at the acute stage. The multivariate logistic regression risk prediction model developed in this study demonstrates good discrimination and calibration, enabling preliminary risk stratification for LVT in patients with acute STEMI; however, further multicenter prospective studies are warranted to validate and refine this model based on the current findings.

## Data Availability

The raw data supporting the conclusions of this article will be made available by the authors, without undue reservation.
